# Thromboprophylaxis in lower limb varicose vein surgery in Brazil

**DOI:** 10.1590/1677-5449.202101721

**Published:** 2022-05-16

**Authors:** Alcides José Araújo Ribeiro, Daniel Mendes-Pinto, Fabiano Luiz Erzinger, Rossano Kepler Alvim Fiorelli, Stênio Karlos Alvim Fiorelli, Andrea Campos de Oliveira Ribeiro, Marcos Arêas Marques

**Affiliations:** 1 Hospital de Base do Distrito Federal, Brasília, DF, Brasil.; 2 Hospital Felício Rocho, Belo Horizonte, MG, Brasil.; 3 Hospital Erasto Gaertner, Curitiba, PR, Brasil.; 4 Universidade Federal do Estado do Rio de Janeiro – UNIRIO, Rio de Janeiro, RJ, Brasil.; 5 Clínica Villas Boas, DF, Brasília, Brasil.; 6 Universidade do Estado do Rio de Janeiro – UERJ, Rio de Janeiro, RJ, Brasil.

**Keywords:** varicose veins, venous thrombosis, pulmonary embolism, patient safety, health services research, prophylaxis

## Abstract

**Background:**

Despite all the investment in primary venous thromboembolism (VTE) prophylaxis for surgical patients in recent years, there are still no specific guidelines for those who undergo procedures to treat lower limb varicose veins.

**Objectives:**

To evaluate the profile of VTE prophylaxis practices among Brazilian vascular surgeons conducting lower limb varicose vein procedures.

**Methods:**

Survey design, sending an electronic questionnaire to Brazilian vascular surgeons. Respondents were divided between those who perform saphenous vein treatment with conventional surgery and those who perform thermoablation for the purpose of comparison between groups.

**Results:**

Of 765 respondents, 405 (53%) treat saphenous veins with conventional surgery for, 44 (6%) with foam, and 199 (26%) with thermoablation (endolaser or radiofrequency). Surgeons who perform thermoablation prescribed more pharmacoprophylaxis after varicose vein surgery than those who perform conventional surgery (67/199, 34% vs. 112/405, 28%; p = 0.002). The thermoablation group stratifies patients for thromboembolism risk more frequently than the conventional surgery group (102/199, 51% vs. 179/405, 44%; p = 0.004). Both groups use enoxaparin as the most frequent drug for prophylaxis, but the thermoablation group uses proportionally more direct oral anticoagulants than the conventional surgery group (26% vs. 10%, p<0.001).

**Conclusions:**

Brazilian vascular surgeons who perform saphenous vein treatment by thermoablation prescribe pharmacoprophylaxis more frequently and for a longer period than those who use conventional surgery.

## INTRODUCTION

Despite all the investment in development of primary prophylaxis to prevent venous thromboembolism (VTE) in clinical and surgical patients over recent decades, there are still no guidelines specifically for those who undergo procedures to treat lower limb (LL) varicose veins.

Prophylaxis to prevent VTE is essential to protect patients who undergo any type of procedure and, among other factors, its efficacy is related to identification of those at greater risk of developing VTE and the type of procedure they will be subjected to.^1^

The true prevalence of VTE associated with the various different treatments for LL varicose veins is still unknown, varying according to the procedure employed: 0.4 to 5.3% for conventional surgery; 0.7 to 16% for radiofrequency ablation; 1% for endovenous laser ablation; and 1 to 3% for ultrasound-guided foam sclerotherapy.^2^^-^7 However, the risk does exist and can remain for up to 1 year after the procedure.^2^^,^^4^^-^7 When a VTE episode occurs within 1 month of the procedure, it is probably more related to an individual risk factor than to the procedure itself.^2^ The range of options available has increased with the advent of less invasive techniques, but the VTE prevalence rates associated with these procedures remain unknown.

In view of the above, the objective of this study was to trace the profile of VTE primary prophylaxis management by vascular surgeons who perform venous procedures in Brazil. The primary objective was to conduct a descriptive analysis of thromboprophylaxis practices employed by Brazilian vascular surgeons. The secondary objective was to analyze differences in thromboprophylaxis practices between a group using conventional surgery and a group using endovenous treatment techniques.

## METHODS

A cross-sectional study was conducted with simple probabilistic sampling. The project was granted approval by the Ethics Committee under decision number 3.966.583.

From July to September of 2019, electronic questionnaires were sent to all Brazilian vascular surgeons and angiologists registered with the Brazilian Society of Angiology and Vascular Surgery (SBACV - Sociedade Brasileira de Angiologia e de Cirurgia Vascular), a total of 3,766 people when the survey was conducted. Additionally, during the same period, questionnaires were also sent via social networks to around 1,500 members of a WhatsApp^®^ group (“Fórum Vascular^®^”) made up of Brazilian angiologists and vascular surgeons, since some of the members of this group are professionals who are not members of the SBACV. There were no exclusion criteria.

Data were collected using an electronic questionnaire compiled using Google Forms^®^. The form contained 29 questions related to venous procedures, based on prior literature on the subject.

The responses to the questions were distributed by frequency. For comparisons between groups, respondents were divided into those who used conventional surgery to treat varicose veins and those who used thermal ablation, defined as treatment of varicose veins using endolaser or radiofrequency. Comparisons of frequencies between these two groups were made using the chi-square test of tendencies or Fisher’s exact test and the significance level adopted was 0.05%. Tabulation and analyses of data were performed in a Microsoft Excel^®^ spreadsheet and with Minitab^®^ version 18 and GraphPad Prism^®^ version 8.

## RESULTS

Questionnaires were sent to approximately 4,000 Brazilian vascular surgeons, 765 of whom responded (approximately 20%). The majority of the respondents performed from one to three varicose vein operations per week (532 [70%]); 209 (27%) performed from four to nine per week; and 19 (3%) reported operating on ten or more cases per week.

**Figure 1:**
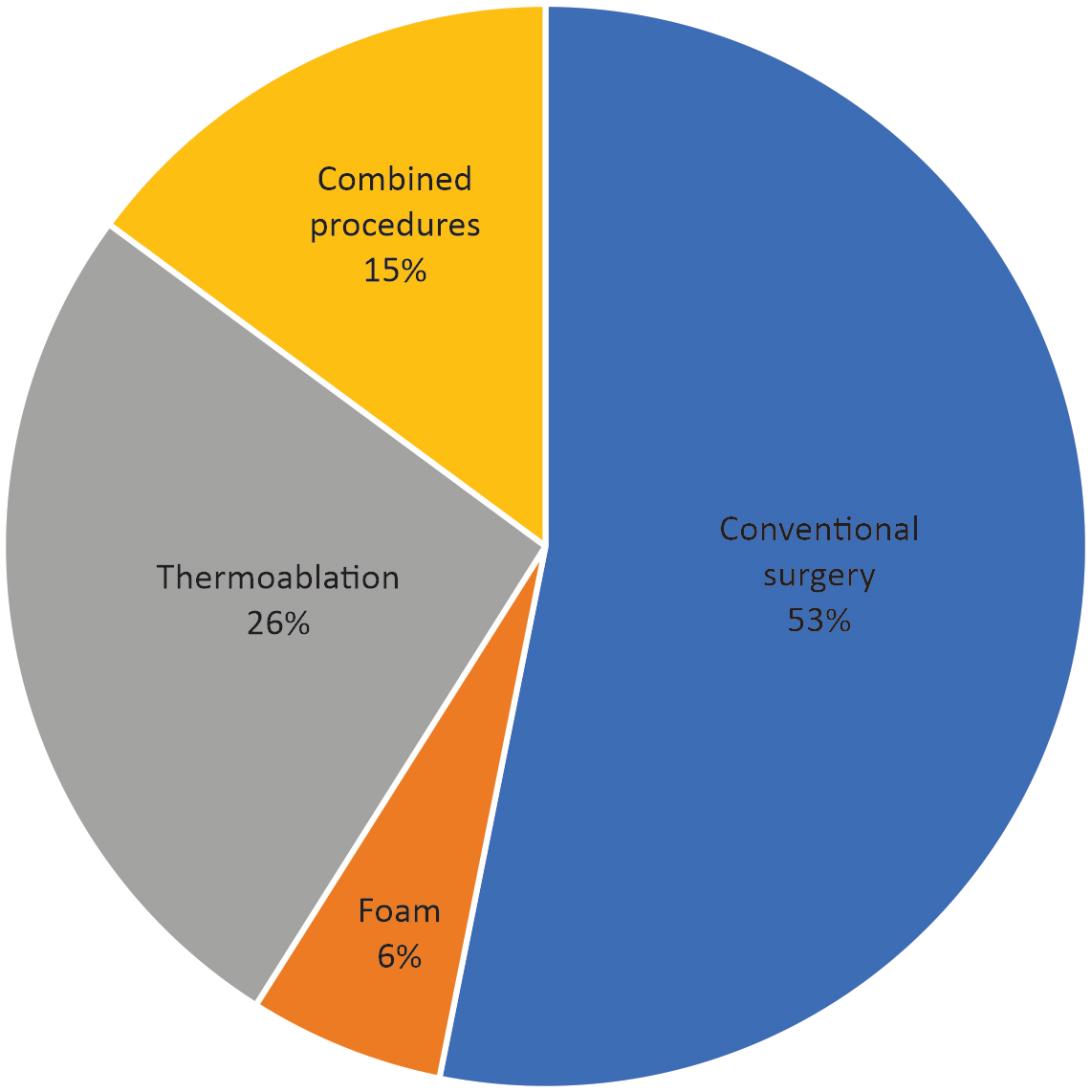
Distribution of preferred treatment for saphenous veins among 765 vascular surgeons.

The preferred treatment for saphenous veins was conventional surgery for 405 (53%) respondents, ultrasound-guided foam sclerotherapy for 44 (6%), thermoablation for 199 (26%), and combined treatment using multiple methods for 113 (15%) (Figure 1).

With regard to preferred thermoablation methods, 319 respondents use endolaser (64%), 153 use radiofrequency (31%), and 25 (5%) use both with equal frequency.

When asked about performing phlebectomies, 722 (95%) respondents stated they performed this treatment concomitantly with saphenous vein treatment, 32 (4%) stated that they perform phlebectomies occasionally, and 5 (1%) replied that they did not perform them.

Table 1 shows the distribution of responses to questions about surgical treatment of saphenous veins between the conventional surgery and thermoablation groups. The most frequent type of anesthesia was spinal anesthesia in both groups (96% in the conventional surgery group and 78% in the thermoablation group); local anesthesia was used more frequently in the thermoablation group (13% vs. 3%), which was a significant difference between the groups (p < 0.001). The most common setting for performing surgery was hospital (96% in the surgery group and 97% in the thermoablation group; p = 0.98). Duplex ultrasound was used during the postoperative period more frequently in the thermoablation group than in the conventional surgery group (4% of the conventional surgery group always order this examination vs. 22% who always order it in the thermoablation group).

**Table 1:** Characteristics of preferred saphenous treatment groups.

	**Conventional surgery group (405)**	**% (CI)**	**Thermoablation group (199)**	**% (CI)**	*p*-value^*^
**What type of anesthesia is used most?**					
Spinal block	388	96% (94;98)	155	78% (72;84)	< 0.001
Local	12	3% (1;5)	26	13% (8;18)	
General	1	0%	3	2% (0;4)	
**In what setting do you routinely conduct varicose veins surgery?**					
Hospital	388	96% (94;98)	193	97% (95;99)	0.98
Clinic	12	4% (2;6)	3	2% (0;4)	
Office	0	0%	2	1% (0;2)	
**Do you order color duplex US during the postoperative period?**					
No	128	32% (27;37)	22	11% (7;15)	< 0.001
Only in selected cases	262	65% (60;70)	133	67% (60;74)	
Always	15	4% (2;6)	44	22% (16;28)	

Values are percentages and 95% confidence intervals (CI).

* Chi-square test of tendencies. US: ultrasonography.

**Figure 2:**
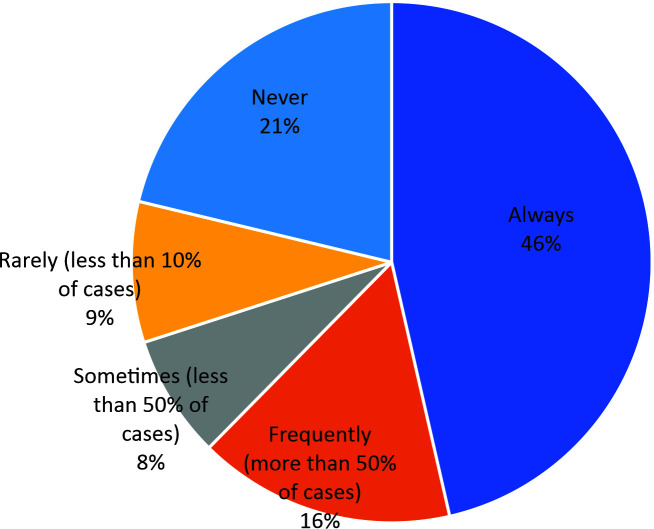
Distribution of prescription of pharmacological prophylaxis after varicose veins surgery.

Taking both conventional surgery and thermal ablation groups together, 30% of the respondents choose to always use pharmacological prophylaxis, 22% rarely use it, 15% use it frequently, 14% sometimes use it, and 18% never prescribe it (Figure 2).

The thermoablation group exhibited a statistically significant tendency to conduct VTE risk stratification and to prescribe pharmacological prophylaxis after varicose veins surgery more often than the conventional surgery group (Table 2). One hundred and twelve respondents (28%) always prescribe pharmacological prophylaxis during the postoperative period in the conventional surgery group vs. 67 (34%) in the thermoablation group (p = 0.002). A higher percentage do not prescribe it in the conventional surgery group (23%) than in the thermoablation group (10%). The most frequently used postoperative prophylaxis drug was enoxaparin in both the conventional surgery group (90%) and the thermoablation group (74%); although the second of these used direct oral anticoagulants (DOAC) with greater frequency (26% in the thermoablation group vs. 10% in the conventional surgery group). There was no difference between the groups in terms of enoxaparin dosages, with 40 mg once a day the preferred dose. In both groups, rivaroxaban was the DOAC most often used. There was a wide range of variation in the duration of pharmacological prophylaxis prescriptions during the postoperative period, with a statistically significant tendency to longer use (7 to 10 days) in the thermoablation group (41% vs. 27% in the conventional surgery group). It was notable that 50% of the conventional surgery group and 40% of the thermoablation group prescribed postoperative prophylaxis for 1 day, while 65% and 49% of the surgeons respectively gave responses within the range of 1 to 3 days.

**Table 2:** Pharmacological prophylaxis practices by preferred saphenous treatment group.

	**Conventional surgery group (405)**	**% (CI)**	**Thermoablation group (199)**	**% (CI)**	*p*-value^*^
**Do you prescribe pharmacological prophylaxis after varicose veins surgery?**					
Always	112	28% (24;32)	67	34% (27;41)	0.002
Frequently (more than 50% of cases)	60	15% (12;18)	32	16% (11;21)	
Sometimes (less than 50% of cases)	50	12% (9;15)	36	18% (13;23)	
Rarely (less than 10% of cases)	88	22% (18;26)	45	23% (17;29)	
No	94	23% (19;27)	19	10% (6;14)	
**If you prescribe pharmacological prophylaxis, which drug class do you use most?**					
Enoxaparin	289	90% (87;93)	151	74% (68;80)	< 0.001
DOACs	31	10% (7;13)	52	26% (20;32)	
Unfractionated heparin	1	0%	0	0%	
**If you prescribe enoxaparin for postoperative prophylaxis, what dose do you use?**					
20 mg/d	46	14% (10;18)	14	8% (4;12)	0.469
40 mg/d	227	71% (66;76)	144	82% (76;88)	
60 mg/d	4	1% (0;2)	4	2% (0;4)	
Undefined	44	14% (10;18)	13	7% (3;11)	
**If you prescribe DOACs for postoperative prophylaxis, which drug do you prefer?**					
Rivaroxaban	174	95% (92;98)	122	95% (91;99)	0.585
Apixaban	7	3% (1;5)	2	1% (0;3)	
Dabigatran	2	1% (0;2)	4	3% (0;6)	
Edoxaban	1	1% (0;2)	1	1% (0;3)	
**For how many days do you prescribe postoperative prophylaxis?**					
1 day	161	50% (45;55)	73	40% (33;47)	0.012
2 days	18	6% (3;9)	9	5% (2;8)	
3 days	28	9% (6;12)	8	4% (1;7)	
5 days	2	1% (0;2)	0	0%	
7 days	63	20% (16;24)	52	29% (22;36)	
10 days	22	7% (4;10)	22	12% (7;17)	
15 days	7	2% (0;4)	4	2% (0;4)	
30 days	2	1% (0;2)	0	0%	
Undefined	19	6% (3;9)	13	7% (3;11)	
**If you do not prescribe pharmacological prophylaxis, what is the main reason?**					
I prescribe compression stockings	68	40% (33;47)	29	48% (35;61)	0.108
Lack of evidence scientific	30	18% (12;24)	17	28% (17;39)	
I prescribe early mobilization	50	29% (22;36)	8	13% (5;21)	
Risk of bleeding	23	13% (8;18)	6	10% (2;18)	
Price	0	0%	1	2% (2;6)	

* Chi-square test of tendencies. Values are percentages and 95% confidence intervals (CI). DOACs: direct oral anticoagulants.

The reasons given for not prescribing pharmacological prophylaxis during the postoperative period did not differ between the groups; the main reason was that patients were prescribed compression stockings. Other reasons were also mentioned, including lack of scientific evidence, encouraging early mobilization, risk of bleeding, and the price of the drugs.

The guidance given by those in the conventional surgery and thermal ablation groups, respectively, to women who were taking oral contraception (OC) before procedure was as follows: 59% and 67% did not instruct them to stop taking OC; 29% and 21% advised stopping 30 days before and resuming 30 days after; 9% and 7% advised stopping less than 30 days before and resuming less than 30 days after; and 2% and 4% instructed patients to stop more than 30 days before and resume more than 30 days after (Table 2).

Table 3 lists the degree of importance attributed to factors related to pharmacological prophylaxis by the two preferred saphenous veins treatment groups. There were no differences between the groups in terms of the degree of importance attributed to the following factors: obesity, relapsed varicose veins, prior history of VTE, bilateral procedures, taking OC, smoking, thrombophilias, family history of VTE, and low mobility. The thermoablation group gave greater importance to cancer than the conventional surgery group (55% vs. 14%; p = 0.048). The thermoablation group classified large varicose veins as of lower importance than the conventional surgery group (16% of the thermoablation group vs. 5% of the conventional surgery group rated them as not very important; p = 0.019).

**Table 3:** Importance of factors related to pharmacological prophylaxis, by preferred saphenous treatment group.

	**Conventional surgery group (405)**	**% (CI)**	**Thermoablation group (199)**	**% (CI)**	*p*-value^*^
**If you prescribe pharmacological prophylaxis selectively, which factors do you consider?**					
**Obesity**					
Very important	90	22% (17;27)	58	29% (22;36)	0.085
Important	169	42% (36;48)	85	43% (35;51)	
Not very important	34	8% (5;11)	12	6% (2;10)	
**Relapsed varicose veins**					
Very important	15	4% (2;6)	8	4% (1;7)	0.315
Important	65	16% (11;21)	45	23% (16;30)	
Not very important	173	43% (37;49)	86	43% (35;51)	
**Prior history of DVT/PE**					
Very important	195	48% (42;54)	112	56% (48;64)	0.064
Important	100	25% (20;30)	46	23% (16;30)	
Not very important	16	4% (2;6)	3	2% (0;4)	
**Bilateral procedures**					
Very important	19	5% (2;8)	12	6% (2;10)	0.76
Important	72	18% (13;23)	34	17% (11;23)	
Not very important	171	42% (36;48)	99	50% (42;58)	
**Oral contraception**					
Very important	75	19% (14;24)	53	27% (20;34)	0.202
Important	144	36% (30;42)	68	34% (26;42)	
Not very important	60	15% (11;19)	31	16% (10;22)	
**Smoking**					
Very important	55	14% (10;18)	39	20% (14;26)	0.284
Important	148	37% (31;43)	73	37% (29;45)	
Not very important	74	18% (13;23)	38	19% (13;25)	
**Thrombophilia**					
Very important	189	47% (41;53)	108	54% (46;62)	0.129
Important	101	25% (20;30)	48	24% (17;31)	
Not very important	16	4% (2;6)	4	2% (0;4)	
**Cancer**					
Very important	185	14% (10;18)	109	55% (47; 63)	0.048
Important	101	32% (27;37)	42	21% (15; 27)	
Not very important	12	23% (18;28)	3	2% (0; 4)	
**Large varicose veins**					
Very important	55	30% (25;35)	37	19% (13;25)	0.019
Important	130	38% (32;44)	82	41% (33;49)	
Not very important	92	5% (2;8)	32	16% (10;22)	
**Family history of DVT/PE**					
Very important	123	32% (27;37)	65	33% (26;40)	0.826
Important	153	35% (30;40)	85	43% (35;51)	
Not very important	20	4% (2;6)	8	4% (1;7)	
**Low mobility**					
Very important	131	32% (27;37)	81	41% (33; 9)	0.313
Important	140	35% (29;41)	68	34% (27;41)	
Not very important	17	4% (2;6)	9	5% (2;8)	

* Chi-square test of tendencies. Values are percentages and 95% confidence intervals (CI). DVT: deep venous thrombosis; PE: pulmonary embolism.

## DISCUSSION

There is still considerable uncertainty about the true incidence of VTE after procedures to treat LL varicose veins and evidence on the need for routine thromboprophylaxis is lacking. The objective of this study was to survey the standard practice of Brazilian vascular surgeons.

Our data show that more than half of the vascular surgeons surveyed employ conventional surgery to treat saphenous veins and around 25% use thermoablation techniques. One of the factors underlying this situation is the fact that thermal ablation, whether by endolaser or by radiofrequency, is a procedure that is not covered by and cannot be billed to the public healthcare system or private health insurance in Brazil. Ultrasound-guided foam sclerotherapy was recently added to the list of varicose veins treatment options available on the Brazilian public healthcare system.

A national survey of vascular surgeons was conducted in Ireland with a 60% response rate, but a low number of participants (30 of 50), in comparison with the present study, which has a larger number of responses (765). When asked about thromboprophylaxis, 73.3% of vascular surgeons replied that they used it routinely and just 6.7% did not use it. The most common reason given for not using thromboprophylaxis was a lack of evidence to support routine use.^8^ With regard to the type of procedures employed, 36.7% of interviewees only used endovenous techniques, 53% used a combination of conventional and endovenous, and 10% only used conventional surgery. Systematic duplex ultrasound was ordered for all patients by 53.3% of the interviewees. The anticoagulants employed were enoxaparin, by 73.3%, or tinzaparin in 23.3% of cases, and 71.4% used a single dose (20 or 40 mg or 3,500 or 4,500 UI, respectively). The authors explained that in a large proportion of the responses, this single dose was used in response to legal issues in an era of defensive medicine because there were so many legal claims involving varicose veins surgery, so this conduct was a form of legal protection. Routine post-procedure duplex ultrasound was used by 23.1% of the surgeons who participated in the survey, but some of these examinations were conducted in an informal manner. Patients are assessed during the postoperative period by 80% of the participants. With regard to OC, 56.7% of the interviewees withdraw them during the perioperative and 26.7% do not. The majority of our interviewees (56.7%) conduct phlebectomies at the same time as the trunk ablation procedure.

All of the interviewees prescribe elastic compression stockings post-ablation, but with variable duration of use. The majority conduct postoperative assessments, but do not routinely use imaging methods.

With regard to VTE, 43.3% state that they know their personal event rates, which vary from 0 to 1% and occur during a follow-up period from 3 to 31 postoperative days. It can be assumed that these rates are for clinically symptomatic VTE, since not all patients routinely undergo post-procedure imaging.

A similar survey conducted in Greece and published in 2012 observed that 52% of interviewees routinely used thromboprophylaxis for conventional varicose veins surgery and 58% for endovenous surgery. Low molecular weight heparin was used almost unanimously (60/63 [95%]) as the preferred pharmacological prophylaxis for conventional surgery (just three surgeons used mechanical methods) and in 100% of endovenous procedures. It should be pointed out that there were no reports of use of other types of heparin or fondaparinux.^9^

In this survey, duration of use of pharmacological prophylaxis was one to two doses in 66% of conventional surgical procedures and in 52% of endovenous procedures.

Just five risk factors were considered to justify use of pharmacological prophylaxis by more than 50% of the vascular surgeons who used it selectively for conventional surgery, as follows: thrombophilias, history of VTE, obesity, history of malignancy, and OC or hormone replacement therapy.

Postoperative duplex ultrasound was used by 48% of surgeons after conventional surgery and by 6% after endovenous procedures. Several different studies describe the lack of consensus on thromboprophylaxis for LL varicose veins surgery and, although this is routine surgery for almost all vascular surgeons, there is still a low but appreciable risk of severe adverse events such as VTE.^8^^–^11

While prevention is considered the best strategy, specific data underscore the need for evidence-based guidelines, since the variability in routine practice can result in medical litigation.

A national survey was conducted in Switzerland of physicians who perform endovenous thermoablation of saphenous veins to assess their thromboprophylaxis practices and their post-procedural follow-up protocols.^10^ Of a total of 121 interviewees, 94 (77.7%) stated that they always or almost always administer pharmacological prophylaxis after thermoablation. One interesting finding of this study was the wide range of variation in duration of pharmacological prophylaxis. Five (4.1%) interviewees stated they prescribe it for just 1 day, while three (3.3%) use prophylaxis for 21 days. However, the majority (57 [47%]) used it for 7 to 10 days,^10^ demonstrating the difficulty of reaching consensus.

The timing of the first dose is a controversial subject in the literature, varying widely. In the Swiss study, 10 physicians (8.3%) chose the response: “start preoperatively 30 minutes to 24 h before intervention”. Sixty-five (53.7%) chose “immediately after the intervention”, and 41 (33.9%) responded “start 1 to 10 hours after intervention”. Two physicians (1.7%) replied that they administer anticoagulant therapy “the day after the intervention”.^10^ In the present study, 85.1% of the Brazilian surgeons start pharmacological prophylaxis during the immediate postoperative period, 10.8%, on the first postoperative day, and the remainder at a variety of different times, ranging from 12 h before surgery (0.3%) to the third postoperative day (0.3%).

An older survey, from 1995, conducted by the Vascular Surgical Society of Great Britain and Ireland, showed that just 12% of vascular surgeons were routinely prescribing pharmacological prophylaxis after conventional surgery. As the years pass, it appears that a growing proportion of surgeons are using prophylaxis, despite the lack of evidence.^12^

To our knowledge, this is the only study conducted in Brazil that has surveyed the VTE prophylaxis profile after procedures to treat LL varicose veins. Comparing it with similar international studies, this is the study with the largest sample that has been published to date.

However, the survey has some limitations. The exact number of people to whom the electronic questionnaire was sent cannot be determined with precision, because it was simultaneously distributed officially to members of the SBACV and sent out via a social network (Fórum Vascular^®^, WhatsApp^®^) and several professionals received the questionnaire by both routes.

Brazil is a country with a very large geographic area and heterogeneous demographics, but sociodemographic data were not collected on the places where the surgeons who responded work. It is probable that pharmacological prophylaxis practices among vascular surgeons in more developed regions are different from those of surgeons in less developed regions. Use of open-ended questions in the questionnaire made it necessary to group responses into several categories and conduct statistical analysis by tendency. However, this approach was important to enable the authors to understand the needs of Brazilian vascular surgeons.

Notwithstanding these limitations, we believe that this initial survey of Brazilian vascular surgeons’ practice is an important foundation to guide public policies and local guidelines on VTE prophylaxis.

## CONCLUSIONS

This study showed that VTE risk assessment and use of and type of pharmacological prophylaxis after procedures to treat LL varicose veins is not uniform among Brazilian vascular surgeons. Those who perform treatment using thermoablation techniques exhibited a greater tendency to perform VTE risk stratification and to prescribe pharmacological prophylaxis and also prescribed it for longer periods.
