# Supplementary material for: Thromboprophylaxis in lower limb varicose vein surgery in Brazil
Source: J Vasc Bras. 2022 May 16;21:e20210172. doi: 10.1590/1677-5449.202101721 (PMC9136684; doi:10.1590/1677-5449.202101721)
Supplement: Translation

# Translation: Tromboprofilaxia na cirurgia de varizes dos membros inferiores no Brasil

**DOI:** 10.1590/1677-5449.202101722

**Authors:** Alcides José Araújo Ribeiro, Daniel Mendes-Pinto, Fabiano Luiz Erzinger, Rossano Kepler Alvim Fiorelli, Stênio Karlos Alvim Fiorelli, Andrea Campos de Oliveira Ribeiro, Marcos Arêas Marques

**Affiliations:** 1 Hospital de Base do Distrito Federal, Brasília, DF, Brasil.; 2 Hospital Felício Rocho, Belo Horizonte, MG, Brasil.; 3 Hospital Erasto Gaertner, Curitiba, PR, Brasil.; 4 Universidade Federal do Estado do Rio de Janeiro – UNIRIO, Rio de Janeiro, RJ, Brasil.; 5 Clínica Villas Boas, DF, Brasília, Brasil.; 6 Universidade do Estado do Rio de Janeiro – UERJ, Rio de Janeiro, RJ, Brasil.

**Keywords:** varizes, trombose venosa, embolia pulmonar, segurança do paciente, pesquisa sobre serviços de saúde, profilaxia

## Abstract

**Contexto:**

Apesar de todo o investimento na profilaxia primária do tromboembolismo venoso (TEV) em pacientes cirúrgicos nos últimos anos, ainda não existem diretrizes específicas para aqueles que serão submetidos a procedimentos para tratamento de varizes de membros inferiores.

**Objetivos:**

Avaliar o perfil de conduta de profilaxia do TEV pelos cirurgiões vasculares brasileiros para procedimentos de tratamento de varizes de membros inferiores.

**Métodos:**

Pesquisa de levantamento por envio de questionário eletrônico a cirurgiões vasculares brasileiros. Os respondentes foram divididos entre os que realizam tratamento de veias safenas por cirurgia convencional e os que realizam termoablação para fim de comparação entre os grupos.

**Resultados:**

Entre os 765 respondentes, o tratamento de escolha das veias safenas foi a cirurgia convencional para 405 (53%), espuma ecoguiada para 44 (6%) e termoablação (*endolaser* ou radiofrequência) para 199 (26%). Os cirurgiões que realizam termoablação prescrevem mais farmacoprofilaxia após o procedimento que aqueles que preferem cirurgia convencional (67/199, 34% vs. 112/405, 28%; p = 0,002). O grupo termoablação estratifica o paciente quanto ao risco de TEV com mais frequência que o grupo cirurgia convencional (102/199, 51% vs. 179/405, 44%; p =0,004). Ambos os grupos usam mais frequentemente enoxaparina como medicação para profilaxia, porém o grupo termoablação usa mais anticoagulantes orais diretos proporcionalmente que o grupo cirurgia convencional (26% vs. 10%, p < 0,001).

**Conclusões:**

Cirurgiões vasculares brasileiros que fizeram o tratamento de veias safenas por termoablação prescrevem farmacoprofilaxia com maior frequência e por um período mais prolongado do que os que realizaram o tratamento por cirurgia convencional.

## INTRODUÇÃO

Apesar de todo o investimento no desenvolvimento da profilaxia primária do tromboembolismo venoso (TEV) em pacientes clínicos e cirúrgicos nas últimas décadas, ainda não existem diretrizes específicas para aqueles que serão submetidos a procedimentos para tratamento de varizes de membros inferiores (MMII).

A profilaxia do TEV é essencial na proteção dos pacientes que serão submetidos a qualquer tipo de procedimento e sua eficácia está relacionada, entre outros fatores, ao reconhecimento daqueles com maior risco de desenvolvê-lo e ao tipo de procedimento aos quais serão submetidos^1^.

A real prevalência do TEV nos diversos tratamentos de varizes de MMII ainda é desconhecida, variando de acordo com o procedimento realizado: 0,4 a 5,3% para a cirurgia convencional; 0,7 a 16% para ablação por radiofrequência; 1% para ablação por *laser* endovenoso; e 1 a 3% para espuma ecoguiada^2^^-^7. Não obstante, esse risco existe e pode persistir por até 1 ano após o procedimento^2^^,^^4^^-^7. Quando o episódio de TEV ocorre em até 1 mês após o procedimento, ele provavelmente está mais relacionado a um fator de risco individual do que ao procedimento propriamente dito^2^. Com o advento de técnicas menos invasivas, as opções se expandiram, mas as prevalências de TEV nesses procedimentos permanece desconhecida.

Diante desses fatos, o objetivo desta pesquisa foi conhecer o perfil das condutas de profilaxia primária do TEV em procedimentos venosos dos cirurgiões vasculares no Brasil. O objetivo principal foi fazer uma análise descritiva das práticas de tromboprofilaxia realizadas por cirurgiões vasculares e angiologistas brasileiros. O objetivo secundário foi analisar diferenças de práticas de tromboprofilaxia entre o grupo de tratamento por cirurgia convencional e o grupo de tratamento por técnicas endovenosas.

## MÉTODOS

Foi realizado um estudo seccional, de amostragem probabilística simples. O projeto obteve aprovação do comitê de ética sob parecer número 3.966.583.

No período de julho a setembro de 2019, foram enviados questionários eletrônicos a todos os cirurgiões vasculares e angiologistas brasileiros associados à Sociedade Brasileira de Angiologia e de Cirurgia Vascular (SBACV), um total de 3.766 na época. Além desses, foram enviados, de forma regular e concomitantemente, questionários via rede social para torno de 1.500 membros do grupo do WhatsApp® (“Fórum Vascular^®^”) composto por angiologistas e cirurgiões vasculares brasileiros, visto que esse grupo incluía também profissionais que não são membros da SBACV. Não houve critério de exclusão.

Os dados foram coletados por meio de envio de questionário eletrônico realizado com o Google Formulários**^®^** . O formulário continha 29 perguntas relacionadas à procedimentos venosos, baseadas em literatura prévia relacionada a esse assunto.

Foi feita a distribuição por frequência das respostas às perguntas. Para fim de comparação entre grupos, os respondentes foram divididos entre aqueles cujo procedimento para tratar varizes é a cirurgia convencional e aqueles que realizam a termoablação, definida pelo tratamento das veias varicosas por *endolaser* ou radiofrequência. A comparação da frequência entre os grupos foi feita pelo teste de qui-quadrado de tendência e exato de Fisher, e o nível de significância estabelecido foi 0,05%. A tabulação de dados e as análises foram feitas em planilha Microsoft Excel® e utilizando os *softwares* Minitab® versão 18 e GraphPad Prism® versão 8.

## RESULTADOS

Foram enviados questionários para aproximadamente 4.000 cirurgiões vasculares brasileiros, e houve resposta de 765 (aproximadamente 20%). A maioria dos respondentes realizam entre uma e três cirurgias de varizes por semana (532 [70%]); 209 (27%) realizam entre quatro e nove cirurgias por semana; e 19 (3%) responderam que operam dez ou mais casos por semana.

O tratamento preferencial das veias safenas foi a cirurgia convencional para 405 (53%) respondentes, espuma ecoguiada para 44 (6%), termoablação para 199 (26%) e o tratamento combinado entre vários métodos para 113 (15%) (Figura 1).

**Figura 1:** Distribuição de preferência para tratamento de veias safenas entre 765 cirurgiões vasculares.

Quanto ao método de termoablação, 319 responderam que realizam *endolaser* (64%), 153 realizam radiofrequência (31%) e 25 (5%) realizam ambos com a mesma frequência.

Quanto à realização de flebectomias, 722 (95%) responderam que realizam tratamento concomitante ao das veias safenas, 32 (4%) responderam que realizam flebectomias ocasionalmente e 5 (1%) responderam que não realizam.

A Tabela 1 mostra a distribuição de questões entre o grupo cirurgia convencional e o grupo termoablação de tratamento cirúrgico das veias safenas. O tipo de anestesia mais utilizada em ambos os grupos foi o bloqueio espinhal (96% no grupo cirurgia convencional e 78% no grupo termoablação); a anestesia local foi mais frequente no grupo termoablação (13% vs. 3%), o que levou a uma diferença significativa entre os grupos (p < 0,001). O local mais comum da realização das cirurgias foi o hospital (96% no grupo cirurgia, 97% no grupo termoablação; p = 0,98). No grupo termoablação, a frequência uso de *duplex scan* no pós-operatório foi maior que no grupo cirurgia convencional (4% solicitam esse exame sempre no grupo cirurgia convencional vs. 22% que solicitam sempre no grupo termoablação).

**Tabela 1:** Características dos grupos de tratamento preferencial das safenas.

	**Grupo cirurgia convencional (405)**	**% (IC)**	**Grupo termoablação (199)**	**% (IC)**	p-valor*
**Qual o tipo de anestesia mais usado?**					
Bloqueio espinhal	388	96% (94;98)	155	78% (72;84)	< 0,001
Local	12	3% (1;5)	26	13% (8;18)	
Geral	1	0%	3	2% (0;4)	
**Em qual local você realiza rotineiramente a cirurgia de varizes?**					
Hospital	388	96% (94;98)	193	97% (95;99)	0,98
Ambulatório	12	4% (2;6)	3	2% (0;4)	
Consultório	0	0%	2	1% (0;2)	
**Você solicita EDC no pós-operatório?**					
Não solicito	128	32% (27;37)	22	11% (7;15)	< 0,001
Somente em casos selecionados	262	65% (60;70)	133	67% (60;74)	
Sempre	15	4% (2;6)	44	22% (16;28)	

Valores expressos em porcentagem e intervalo de confiança (IC) de 95%.

* Teste do qui-quadro de tendência. Valores de porcentagem (%) e intervalo de confiança de 95%. EDC: eco-Doppler colorido.

Considerando-se a cirurgia convencional e a termoablação em conjunto, 30% dos respondentes sempre optam pela farmacoprofilaxia, 22%, raramente, 15%, frequentemente, 14%, às vezes e 18% não a prescrevem (Figura 2).

**Figura 2:** Distribuição quanto à prescrição de farmacoprofilaxia após cirurgia de varizes.

O grupo termoablação teve uma tendência estatisticamente significativa para realizar estratificação de risco para TEV e para prescrição de farmacoprofilaxia após a cirurgia de varizes que o grupo cirurgia convencional (Tabela 2). Cento e doze respondentes (28%) sempre prescrevem farmacoprofilaxia no pós-operatório no grupo cirurgia convencional vs. 67 (34%) do grupo termoablação (p = 0,002). A porcentagem que não prescreve foi maior no grupo cirurgia convencional (23%) que no grupo termoablação (10%). No grupo cirurgia convencional, o fármaco mais comum utilizado na profilaxia pós-operatória foi a enoxaparina (90%), assim como no grupo termoablação (74%); no entanto, neste último, a frequência de uso de anticoagulante oral direto (DOAC) foi maior (26% no grupo termoablação vs. 10% no grupo cirurgia convencional). Não houve diferença entre os grupos quanto à dosagem de uso de enoxaparina, sendo a dose de 40 mg uma vez ao dia a mais frequente. O DOAC mais utilizado foi a rivaroxabana em ambos os grupos. Houve grande variação quanto ao tempo de prescrição da farmacoprofilaxia no pós-operatório, com uma tendência estatisticamente significativa de maior tempo de uso (sete a dez dias) no grupo termoablação (41% vs. 27% no grupo de cirurgia convencional). Chama a atenção o uso por um dia em 50% das respostas do grupo da cirurgia convencional e em 40% no grupo de termoablação; se consideramos de um a três dias, temos um uso por 65% e 49% dos cirurgiões, respectivamente.

**Tabela 2:** Prática de farmacoprofilaxia conforme o grupo de tratamento preferencial das safenas.

	**Grupo cirurgia convencional (405)**	**% (IC)**	**Grupo termoablação (199)**	**% (IC)**	p-valor*
**Você prescreve farmacoprofilaxia após cirurgia de varizes?**					
Sempre	112	28% (24;32)	67	34% (27;41)	0,002
Frequentemente (mais de 50% dos casos)	60	15% (12;18)	32	16% (11;21)	
Às vezes (menos de 50% dos casos)	50	12% (9;15)	36	18% (13;23)	
Raramente (menos de 10% dos casos)	88	22% (18;26)	45	23% (17;29)	
Não prescrevo	94	23% (19;27)	19	10% (6;14)	
**Se você prescreve farmacoprofilaxia, qual classe de fármaco usa mais?**					
Enoxaparina	289	90% (87;93)	151	74% (68;80)	< 0,001
DOACs	31	10% (7;13)	52	26% (20;32)	
Heparina não fracionada	1	0%	0	0%	
**Se você prescreve enoxaparina para profilaxia pós-operatória, qual dose utiliza?**					
20 mg/d	46	14% (10;18)	14	8% (4;12)	0,469
40 mg/d	227	71% (66;76)	144	82% (76;88)	
60 mg/d	4	1% (0;2)	4	2% (0;4)	
Indefinida	44	14% (10;18)	13	7% (3;11)	
**Se você prescreve DOACs para profilaxia pós-operatória, qual medicação prefere?**					
Rivaroxabana	174	95% (92;98)	122	95% (91;99)	0,585
Apixabana	7	3% (1;5)	2	1% (0;3)	
Dabigatrana	2	1% (0;2)	4	3% (0;6)	
Edoxabana	1	1% (0;2)	1	1% (0;3)	
**Por quantos dias você prescreve profilaxia no pós-operatório?**					
1 dia	161	50% (45;55)	73	40% (33;47)	0,012
2 dias	18	6% (3;9)	9	5% (2;8)	
3 dias	28	9% (6;12)	8	4% (1;7)	
5 dias	2	1% (0;2)	0	0%	
7 dias	63	20% (16;24)	52	29% (22;36)	
10 dias	22	7% (4;10)	22	12% (7;17)	
15 dias	7	2% (0;4)	4	2% (0;4)	
30 dias	2	1% (0;2)	0	0%	
Indefinido	19	6% (3;9)	13	7% (3;11)	
**Se você não usa farmacoprofilaxia, qual o principal motivo?**					
Indico meia elástica	68	40% (33;47)	29	48% (35;61)	0,108
Falta de evidência científica	30	18% (12;24)	17	28% (17;39)	
Indico deambulação precoce	50	29% (22;36)	8	13% (5;21)	
Risco de sangramento	23	13% (8;18)	6	10% (2;18)	
Preço	0	0%	1	2% (2;6)	

* Teste do qui-quadrado de tendência. Valores de porcentagem (%) e intervalo de confiança (IC) de 95%. DOACs: anticoagulantes orais diretos.

Quanto ao motivo por não utilizar a farmacoprofilaxia no pós-operatório, não houve diferença de motivo entre os grupos; o motivo principal foi o uso da meia elástica de compressão. Outras medidas foram descritas, como falta de evidência científica, estímulo à deambulação precoce, risco de sangramento e preço da medicação.

Quanto à orientação para as mulheres em relação ao anticoncepcional oral (ACO) antes do procedimento para o grupo da cirurgia convencional e o grupo da termoablação, respectivamente: 59% e 67% não suspendem o seu uso; 29% e 21% suspendem 30 dias antes e voltam 30 dias depois; 9% e 7% suspendem menos de 30 dias e retornam menos de 30 dias depois; e 2% e 4% suspendem mais de 30 dias e retornam mais de 30 dias depois (Tabela 2).

A Tabela 3 mostra o grau de importância de fatores relacionados a farmacoprofilaxia entre os grupos preferenciais de tratamento das veias safenas. Não houve diferença entre os grupos quanto ao grau de importância para os fatores obesidade, varizes recidivadas, história prévia de TEV, procedimentos bilaterais, uso de ACO, tabagismo, trombofilias, história familiar de TEV e pouca mobilidade. O grupo termoablação respondeu que dá mais importância ao fator neoplasia que o grupo cirurgia convencional (55% vs. 14%; p = 0,048). O grupo termoablação classificou com grau menor de importância o fator varizes grandes que o grupo cirurgia convencional (16% consideraram pouco importante no grupo termoablação vs. 5% no grupo cirurgia convencional; p = 0,019).

**Tabela 3:** Importância de fatores relacionados à farmacoprofilaxia entre os grupos de tratamento preferencial da safenas.

	**Grupo cirurgia convencional (405)**	**% (IC)**	**Grupo termoablação (199)**	**% (IC)**	p-valor*
**Se você usa farmacoprofilaxia seletivamente, quais fatores você considera?**					
**Obesidade**					
Muito importante	90	22% (17;27)	58	29% (22;36)	0,085
Importante	169	42% (36;48)	85	43% (35;51)	
Pouco importante	34	8% (5;11)	12	6% (2;10)	
**Varizes recidivadas**					
Muito importante	15	4% (2;6)	8	4% (1;7)	0,315
Importante	65	16% (11;21)	45	23% (16;30)	
Pouco importante	173	43% (37;49)	86	43% (35;51)	
**História prévia de TVP/EP**					
Muito importante	195	48% (42;54)	112	56% (48;64)	0,064
Importante	100	25% (20;30)	46	23% (16;30)	
Pouco importante	16	4% (2;6)	3	2% (0;4)	
**Procedimentos bilaterais**					
Muito importante	19	5% (2;8)	12	6% (2;10)	0,76
Importante	72	18% (13;23)	34	17% (11;23)	
Pouco importante	171	42% (36;48)	99	50% (42;58)	
**Uso de anticoncepcional oral**					
Muito importante	75	19% (14;24)	53	27% (20;34)	0,202
Importante	144	36% (30;42)	68	34% (26;42)	
Pouco importante	60	15% (11;19)	31	16% (10;22)	
**Tabagismo**					
Muito importante	55	14% (10;18)	39	20% (14;26)	0,284
Importante	148	37% (31;43)	73	37% (29;45)	
Pouco importante	74	18% (13;23)	38	19% (13;25)	
**Trombofilia**					
Muito importante	189	47% (41;53)	108	54% (46;62)	0,129
Importante	101	25% (20;30)	48	24% (17;31)	
Pouco importante	16	4% (2;6)	4	2% (0;4)	
**Neoplasia**					
Muito importante	185	14% (10;18)	109	55% (47; 63)	0,048
Importante	101	32% (27;37)	42	21% (15; 27)	
Pouco importante	12	23% (18;28)	3	2% (0; 4)	
**Varizes grandes**					
Muito importante	55	30% (25;35)	37	19% (13;25)	0,019
Importante	130	38% (32;44)	82	41% (33;49)	
Pouco importante	92	5% (2;8)	32	16% (10;22)	
**História familiar de TVP/EP**					
Muito importante	123	32% (27;37)	65	33% (26;40)	0,826
Importante	153	35% (30;40)	85	43% (35;51)	
Pouco importante	20	4% (2;6)	8	4% (1;7)	
**Pouca mobilidade**					
Muito importante	131	32% (27;37)	81	41% (33; 9)	0,313
Importante	140	35% (29;41)	68	34% (27;41)	
Pouco importante	17	4% (2;6)	9	5% (2;8)	

* Teste do qui-quadrado de tendência. Valores de porcentagem (%) e intervalo de confiança (IC) de 95%. TVP: trombose venosa profunda; EP: embolia pulmonar.

## DISCUSSÃO

Ainda existe incerteza considerável sobre a verdadeira incidência de TEV após procedimentos para tratamento de varizes de MMII e uma falta de evidências sobre a necessidade de tromboprofilaxia de rotina. O objetivo desta pesquisa foi avaliar os padrões de prática entre cirurgiões vasculares brasileiros.

Nossos dados mostram que pouco mais da metade dos cirurgiões vasculares realizam a cirurgia convencional para o tratamento das veias safenas e em torno de 25% realizam pelas técnicas de termoablação. Um dos fatores que explica essa condição é o fato de que a termoablação, seja por *endolaser* ou radiofrequência, não é um procedimento reembolsado tanto no sistema público quanto por operadoras de saúde no Brasil. Recentemente, a técnica de escleroterapia por espuma ecoguiada foi incluída nas opções para tratamento de varizes no sistema público brasileiro.

Foi realizada uma enquete nacional entre os cirurgiões vasculares da Irlanda, com taxa de resposta de 60%, mas com poucos participantes (30 de 50), comparando com este estudo, com número de respostas superior (765 respostas). Quando questionados sobre a tromboprofilaxia, 73,3% dos cirurgiões vasculares responderam que a faziam rotineiramente e apenas 6,7% não a realizavam. A razão mais comum apresentada para a não realização da tromboprofilaxia foi a falta de evidências que sustentassem seu uso rotineiro^8^. Com relação ao tipo de procedimento realizado, 36,7% dos entrevistados utilizam apenas técnicas endovenosas, 53% utilizam a combinação convencional e endovenosa e 10% usam somente convencional. O *duplex-scan* sistemático em todos os pacientes é solicitado por 53,3% dos entrevistados. Os anticoagulantes usados foram enoxaparina em 73,3% ou tinzaparina em 23,3% dos casos, e 71,4% utilizaram uma dose única (20 ou 40 mg ou 3.500 ou 4.500 UI, respectivamente). Essa dose única em grande parte das respostas deve-se, segundo os autores, a aspectos legais em época de medicina defensiva e ao fato de haver grande número de processos judiciais envolvendo cirurgia de varizes, o que seria, para muitos, uma forma de proteção jurídica. O *duplex-scan* de rotina pós-procedimento é solicitado por 23,1% dos cirurgiões participantes da enquete, mas parte dos exames é realizada de maneira informal. Os pacientes são avaliados no pós-operatório por 80% dos participantes. Em relação ao ACO, 56,7% dos entrevistados os suspendem no período perioperatório e 26,7% mantêm o seu uso. A maioria de nossos entrevistados (56,7%) realiza flebectomias ao mesmo tempo que o procedimento de ablação troncular.

Todos os entrevistados prescrevem meias elásticas de compressão pós-ablação, mas com durações de uso variáveis. A maioria faz avaliação pós-operatória, mas não com métodos de imagem de rotina.

Em relação ao TEV, 43,3% afirmam conhecer sua taxa pessoal de eventos, que variaram de 0 a 1% e ocorreram em um período de seguimento entre três e 31 dias de pós-operatório. Pode-se presumir que essa taxa represente TEV clinicamente sintomáticos, pois nem todos os pacientes foram submetidos rotineiramente a métodos de imagens pós-procedimento.

Em enquete semelhante, realizada na Grécia e publicada em 2012, observou-se que 52% dos entrevistados utilizavam tromboprofilaxia de rotina em cirurgia de varizes convencional e 58% em cirurgia endovenosa. A heparina de baixo peso molecular (HBPM) foi utilizada quase que unanimemente (60/63 [95%]) como farmacoprofilaxia preferida na cirurgia convencional (apenas três cirurgiões utilizaram métodos mecânicos) e em 100% em procedimentos endovenosos. Cabe ressaltar que não houve relato de uso de outras heparinas ou fondaparinux^9^.

A duração do uso da farmacoprofilaxia, nesta enquete, foi de uma a duas doses em 66% das cirurgias convencionais e em 52% dos procedimentos endovenosos.

Apenas cinco fatores de risco foram considerados para justificar a farmacoprofilaxia por mais de 50% dos cirurgiões vasculares que a utilizavam seletivamente em cirurgia convencional, são eles: trombofilias, história de TEV, obesidade, história de malignidade e uso de ACO ou terapia de reposição hormonal.

O *duplex-scan* no pós-operatório foi realizado por 48% dos cirurgiões após cirurgia convencional e por 6% após procedimento endovenoso. Vários estudos descrevem a falta de consenso na tromboprofilaxia na cirurgia de varizes de MMII, e, embora essa cirurgia seja rotina para quase todos os cirurgiões vasculares, um risco baixo, mas apreciável de eventos adversos graves, como o TEV, ainda existe^8^^-^11. 

Embora a prevenção seja considerada a melhor estratégia, dados específicos reforçam a necessidade de diretrizes baseadas em evidências, pois podem ocorrer complicações médico-legais, considerando a variabilidade na prática cotidiana.

Uma pesquisa nacional entre médicos da Suíça que realizam a termoablação endovenosa de veias safenas foi conduzida para avaliar as suas práticas de tromboprofilaxia e os protocolos de acompanhamento após o procedimento^10^. Dos 121 entrevistados, 94 (77,7%) relataram que sempre ou quase sempre administram farmacoprofilaxia após termoablação. Um achado interessante dessa pesquisa foi a grande variação na duração da farmacoprofilaxia. Cinco (4,1%) dos entrevistados relataram a utilizar por apenas um dia, enquanto três (3,3%) a utilizam por 21 dias. Porém, a maioria (57 [47%]) a usou por um período de sete a dez dias^10^, demonstrando a dificuldade de consenso.

O momento da primeira dose é um assunto controverso na literatura e varia amplamente. No estudo suíço, a resposta escolhida foi: “início pré-operatório 30 minutos a 24 horas antes da intervenção” por dez médicos (8,3%). A resposta foi “imediatamente após a intervenção” por 65 médicos (53,7%), seguida de “início uma a dez horas após a intervenção” por 41 médicos (33,9%). Dois médicos (1,7%) responderam que administram terapia anticoagulante “no dia após a intervenção”^10^. Neste presente estudo, 85,1% dos cirurgiões brasileiros iniciam a farmacoprofilaxia no pós-operatório imediato, 10,8%, no primeiro dia de pós-operatório e o restante, em momentos variados, incluindo desde 12 h antes da cirurgia (0,3%) até o terceiro dia de pós-operatório (0,3%).

Uma pesquisa mais antiga, de 1995, da Sociedade Cirúrgica Vascular da Grã-Bretanha e Irlanda mostrou que apenas 12% dos cirurgiões vasculares prescreviam farmacoprofilaxia de rotina após cirurgia convencional. Com o passar do tempo, um número crescente de cirurgiões parece estar fazendo profilaxia, apesar da falta de evidências^12^.

Pelo nosso conhecimento, este é o único estudo de levantamento sobre o perfil de profilaxia para TEV após procedimentos para tratamento de varizes de MMII realizado no Brasil. Entre estudos internacionais semelhantes, este é o de maior casuística publicado até o momento.

Entretanto, algumas são as limitações deste levantamento. O número exato de pessoas para que o questionário eletrônico foi enviado não pôde ser determinado com precisão, devido à disseminação do mesmo oficialmente aos membros da SBACV, concomitantemente via rede social (Fórum Vascular**^®^** , WhatsApp**^®^** ), e vários profissionais receberam o questionário pelas duas vias.

O Brasil é um país de grande extensão territorial e com demografia heterogênea; dados sociodemográficos relativos ao local de trabalho dos cirurgiões respondentes não foram coletados. É provável que a conduta de farmacoprofilaxia entre cirurgiões vasculares de regiões mais desenvolvidas seja diferente daquela de regiões menos desenvolvidas. A utilização de respostas abertas no questionário levou à necessidade de respostas em várias categorias e de avaliação estatística por tendência; por outro lado, esse levantamento foi importante para que os autores tenham conhecimento das necessidades dos cirurgiões vasculares brasileiros.

Mesmo com essas limitações, acreditamos que este levantamento inicial sobre a conduta dos cirurgiões vasculares brasileiros será importante como base para orientação de políticas públicas e diretrizes locais sobre profilaxia do TEV.

## CONCLUSÃO

Este estudo mostrou que a avaliação de risco para TEV, do uso e do tipo de farmacoprofilaxia após procedimentos para o tratamento de varizes de MMII foi feita de maneira heterogênea pelos cirurgiões vasculares brasileiros. O grupo que realizou as cirurgias por técnicas de termoablação teve maior tendência a fazer a estratificação de risco do TEV e a farmacoprofilaxia, bem como prescrevê-la por um período maior.
